# The Impact of Hyperglycemic Emergencies on the Kidney and Liver

**DOI:** 10.1155/2013/967097

**Published:** 2013-10-24

**Authors:** Feng Bai, Fang-fang Jiang, Jun-jie Lu, Shao-gang Ma, Yi-gen Peng, Yue Jin, Wei Xu, Jian-ping Cheng, Hai-feng Wu

**Affiliations:** ^1^Department of Endocrinology and Metabolism, Huai'an Hospital Affiliated to Xuzhou Medical College and Huai'an Second People's Hospital, Huai'an 223002, China; ^2^Department of Endocrinology and Metabolism, The Third Hospital Affiliated to Nanchang University, Nanchang, Jiangxi 330008, China; ^3^Department of Critical Care Medicine, The Affiliated Yixing People's Hospital of Jiangsu University, No. 75 Tong Zhenguan Road, Yixing, Jiangsu 214200, China; ^4^Department of Emergency, Huai'an Hospital Affiliated to Xuzhou Medical College and Huai'an Second People's Hospital, Huai'an 223002, China; ^5^Department of Clinical Laboratory, Huai'an Hospital Affiliated to Xuzhou Medical College and Huai'an Second People's Hospital, Huai'an, Jiangsu 223002, China

## Abstract

Studies on the alterations of liver and kidney function parameters in patients with diabetic ketoacidosis (DKA) and diabetic ketosis (DK) were limited. Participants with DKA, DK, non-DK, and healthy controls were enrolled in the current study. Parameters of liver and kidney function were measured and evaluated. The patients with DKA had higher levels of plasma glucose, hemoglobin A1c (HbA1c), uric acid, and creatinine but lower levels of transferases and protein compared with the other three groups (*P* < 0.05 for all). The patients with DK had higher levels of plasma glucose and HbA1c but lower levels of glutamyl transpeptidase and protein compared with the non-DK and control groups (*P* < 0.05). Prealbumin levels were significantly reduced in the severe DKA patients compared with the mild/moderate DKA patients. Serum prealbumin levels were correlated with albumin levels (*r* = 0.401, *P* = 0.010), HCO_3_ (*r* = 0.350, *P* = 0.027), and arterial pH (*r* = 0.597, *P* < 0.001) in the DKA patients. A diagnostic analysis showed that lower prealbumin levels significantly reflected the presence of hyperglycemic emergencies (*P* < 0.001). Liver and kidney function parameters deteriorated, especially in DKA. Prealbumin levels can be of value in detecting the presence of hyperglycemic crisis. This clinical trial is registered with ChiCTR-OCH-12003077.

## 1. Introduction

Diabetic ketoacidosis (DKA) and diabetic ketosis (DK) are common and serious complications of diabetes mellitus. DKA most often occurs in patients with type 1 diabetes (T1D). However, increasing evidence indicates that DKA and DK are also common features of ketosis-prone type 2 diabetes (T2D) [1]. In addition, patients with T2D are susceptible to DKA under stressful and infectious conditions. DKA in patients with T2D is a more severe disease, with worse outcomes, compared with concomitant DKA and T1D [2]. However, DKA patients with either T2D or T1D require timely treatment. Delays in the diagnosis and treatment of DKA can lead to increased risks of advanced complications and 30-day mortality [3].

Previous studies in the field of DKA have paid much attention to proinflammatory cytokines, whereas clinical parameters of liver and kidney function have not been well characterized [4, 5]. Tests of hepatic function include assessments of albumin, prealbumin, bilirubin, and enzyme levels. Increased levels of aspartate aminotransferase (AST), alanine aminotransferase (ALT), and *γ*-glutamyl transferase (GGT) are associated with T2D incidence [6, 7]. Few studies have focused on the detection of these reliable molecules. These parameters are routinely detected in diabetic emergencies but do not receive attention from clinicians. Elevations of serum creatinine, uric acid, and potassium levels have been observed in patients with DKA [8, 9]. The influence of DKA and DK on liver and kidney function-related variables has not been well characterized and is thus poorly understood.

Prealbumin, a marker for protein malnutrition, has been advocated as a useful marker for predicting gastric surgery complications, cardiovascular outcome, and mortality in diabetic hemodialysis patients [10, 11]. To our knowledge, there is no available study on the patterns or prognostic value of prealbumin levels in patients with DKA and DK. Accordingly, we aimed to evaluate changes in prealbumin levels and their diagnostic value in reflecting the severity of hyperglycemic emergencies. It should be stressed that previous studies only provided limited information and did not characterize the correlation with hyperglycemic crises [5, 10, 11]. The current study was performed due to limitations of the existing data in the literature. In particular, this study addressed whether kidney function increases or hepatic function decreases. The detection of kidney-liver function parameters will be helpful not only for early assessment but also for determining the prognosis of diabetes mellitus with hyperglycemic crisis.

## 2. Methods

### 2.1. Patient Recruitment and Exclusion Criteria

This study was performed in the endocrinology and emergency departments of two medical college-affiliated hospitals from October 2012 to March 2013 in accordance with the principles of the Declaration of Helsinki (2001). Ethical approval was obtained from the Ethical Committees of Xuzhou Medical College and Nanchang University. Written informed consent was obtained from all of the participants.

A total of 40 consecutive patients with DKA (DKA group), 40 patients with DK (DK group), 40 diabetic patients in stable condition (non-DK group), and 40 normal control subjects (control group) participated in the study (Table 1). The DKA group included 19 T1D and 21 T2D patients. Patients with DKA had a plasma glucose level > 13.90 mmol/L, a urine ketone level defined as moderate to high (+ to +++), and an arterial pH value < 7.30 at the time of admission. The criteria for DKA severity were as follows, as previously described: mild, 7.20 ≤ pH < 7.30; moderate, 7.10 ≤ pH < 7.20; and severe, pH < 7.10 [4, 12, 13].

The participants underwent a routine medical examination. None of the subjects had heart failure, hematologic disease, or liver or kidney dysfunction. Their previous liver or kidney dysfunctions were within normal range, and none of the patients were taking steroids. The non-DK and control subjects did not have fatty livers. The DKA, DK, and non-DK patients received medical treatment in the form of nutrition, oral hypoglycemic agents, and insulin therapy. Patients with DKA and DK were monitored until their ketosis and/or acidosis resolved.

### 2.2. Biochemical Assays

Blood samples from the control and non-DK groups were collected into Vacutainer tubes by venipuncture after an overnight fast. In the DKA and DK groups, the blood samples were drawn both at admission, before the initial therapy, and at the time of discharge from the hospital.

Laboratory tests, including routine biochemistry tests, were performed using routine laboratory methods for such serum parameters as albumin, ALT, AST, bilirubin, creatinine, prealbumin, and uric acid levels. Blood samples were collected into tubes for kidney-liver function tests and immediately processed using an Olympus AU 2700 autoanalyzer (Olympus, Tokyo, Japan). Arterial gas analyses were performed using commercial kits and a GEM Premier 3000 (Instrumentation Laboratory, USA). Glycated hemoglobin A1c (HbA1c) levels were measured using an HbA1c meter from Bio-Rad Laboratories, Ltd. (Shanghai, China). The estimated Glomerular Filtration Rate (eGFR) was calculated by using the estimating equation from Chinese patients with CKD [14].

### 2.3. Statistical Methods

The results are expressed as the mean ± SD for quantitative variables with normal distributions. Skewed parameters are presented as the median and range (min–max). Pairwise comparisons of the four groups were performed using the Tukey and Kruskal-Wallis nonparametric ANOVA tests. A Mann-Whitney U analysis was used as the nonparametric test for the two subgroups. Chi-squared tests were utilized for the comparison of other clinical features. Correlations between the observed variables were analyzed by Pearson's correlation test. The risk markers for the diagnosis of DKA and DK were assessed by multiple logistic regression analyses. Receiver operating characteristic (ROC) curve analyses were performed to determine the diagnostic performance of each variable. Statistical analyses were conducted with the SPSS 18.0 software (SPSS Inc., Chicago, IL) and MedCalc version 12.1.4.0. A two-tailed *P* value < 0.05 was considered statistically significant.

## 3. Results

### 3.1. Baseline Clinical and Laboratory Characteristics

The baseline demographic and clinical characteristics of the subjects are shown in Table 1. A total of 160 cases were included in this study. Regarding age, gender, and diabetic type, there were no significant differences. There were 15 patients with mild DKA, 10 patients with moderate DKA, and 15 patients with severe DKA. The glycemic variables (plasma glucose and HbA1c levels) had significantly higher values in the three diabetic patient groups than in the control group (*P* < 0.001). The glycemic variables had higher values in the DKA and DK groups than in the non-DK group (*P* < 0.05). The difference in the duration of diabetes reached statistical significance (*P* < 0.05).

The differences in the kidney-liver function values among the four groups were statistically significant (*P* < 0.05). This study demonstrated that most of the liver function-related concentrations were significantly lower in DKA patients compared with the other three groups (*P* < 0.001 or *P* < 0.05). Serum creatinine and uric acid levels were significantly elevated in the DKA patients (*P* < 0.05). Accordingly, the eGFR levels were significantly elevated in the DKA patients (*P* < 0.05). However, the levels of AST and bilirubin were not different among the four groups. Of all of the liver function parameters in DK patients, the levels of GGT, total protein, albumin, globulin, and prealbumin were significantly lower than in patients with stable diabetes and control subjects (*P* < 0.001 and *P* < 0.05, resp.). These results are shown in Table 1.

As shown in Table 2, the DKA patients were divided into T1D and T2D subgroups. Plasma glucose levels at the time of admission were significantly higher in the patients with T1D compared with the patients with T2D (31.82 ± 8.53 mmol/L versus 26.37 ± 6.81 mmol/L, *P* = 0.035). In addition, the patients with T2D were older than the patients with T1D (35 ± 19 years versus 52 ± 10 years, *P* = 0.001). The levels of prealbumin were similar between the T1D and the T2D cases (*P* = 0.775). No significant differences were observed regarding the other parameters.

As in Table 3, the DK and DKA patients were divided into infection and noninfection subgroups, respectively. The infection subgroup contained more female cases and older individuals (*P* < 0.05). Among DK patients, the infection subgroup had significantly lower levels of albumin and ALT (*P* = 0.016 and *P* = 0.001, resp.). However, among DKA patients, protein levels related to kidney-liver function were similar in both subgroups. Furthermore, there was no difference in the prealbumin levels of the two subgroups.

As shown in Table 4, there were 15 patients with severe DKA, 10 patients with moderate DKA, and 15 patients with mild DKA. The levels of arterial pH, HCO_3_, and prealbumin were significantly lower in the severe DKA patients compared with the mild/moderate DKA patients (*P* < 0.05). For the rest of the variables, there were no significant differences between the mild/moderate and severe DKA cases.

When the DKA and DK resolved after treatment, the levels of HCO_3_, arterial pH, and plasma glucose returned to normal. Accordingly, the kidney function parameters declined to the normal range, but liver function-related levels were higher than at admission (all *P* < 0.05, Table 1).

### 3.2. Correlation and Regression Analyses

**Figure 1:**
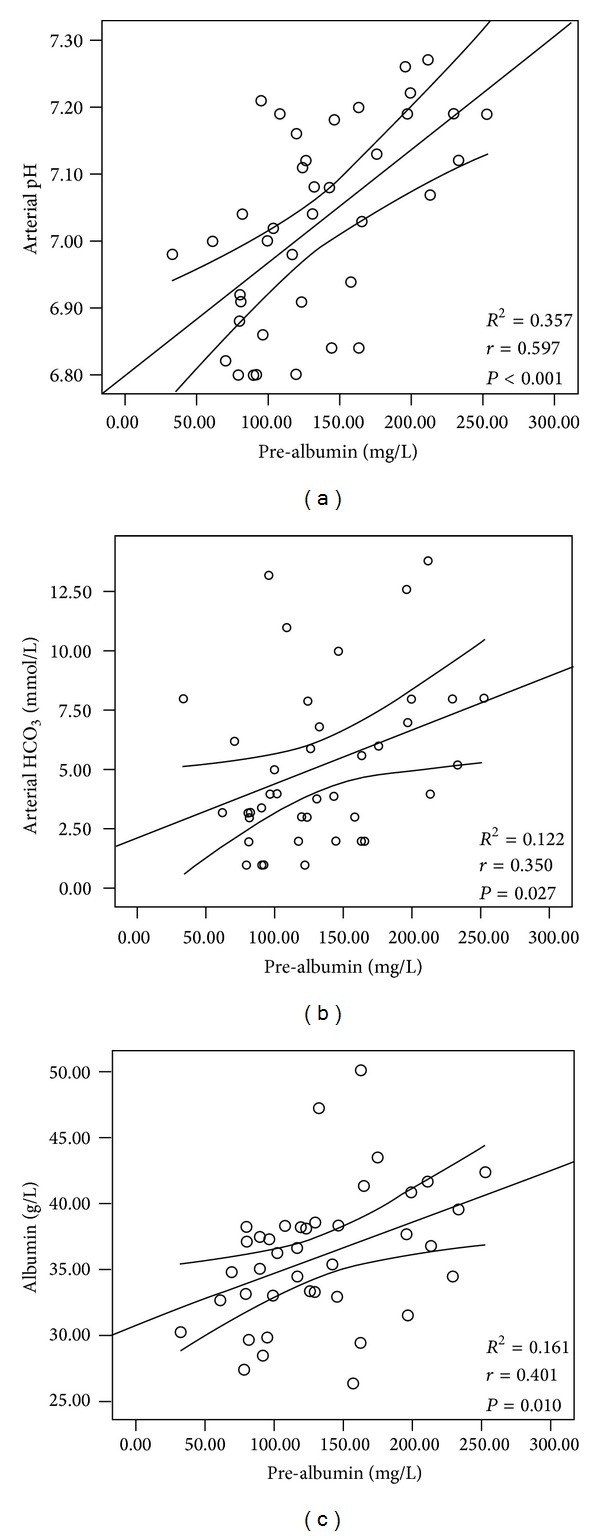
Serum prealbumin levels were positively correlated with the levels of albumin ((a): *r* = 0.401, *P* = 0.010), HCO_3_ ((b): *r* = 0.350, *P* = 0.027), and arterial pH ((c): *r* = 0.597, *P* < 0.001) in patients with DKA.

Bivariate correlation analyses were performed to assess the relationships at baseline in DKA and DK patients (Figure 1). In DKA patients, serum prealbumin levels were positively correlated with albumin levels (Figure 1(a): *r* = 0.401, *P* = 0.010), HCO_3_ levels (Figure 1(b): *r* = 0.350, *P* = 0.027), and arterial pH (Figure 1(c): *r* = 0.597, *P* < 0.001). In DK patients, there was no clinical correlation. Accordingly, pH was the most significant factor influencing the prealbumin levels (**β** = 0.768, *P* < 0.001) but the levels of albumin (**β** = 0.292, *P* = 0.024) and HCO_3_ (**β** = −0.299, *P* = 0.129) were not. Multiple logistic regression analyses with significant clinical variables (albumin and prealbumin levels) were performed for the DKA and DK groups. The odds ratios (ORs) and 95% confidence intervals (CIs) were calculated. There were no clinically significant differences in the DKA and the DK groups. The results are not shown.

### 3.3. ROC Analyses

**Figure 2:**
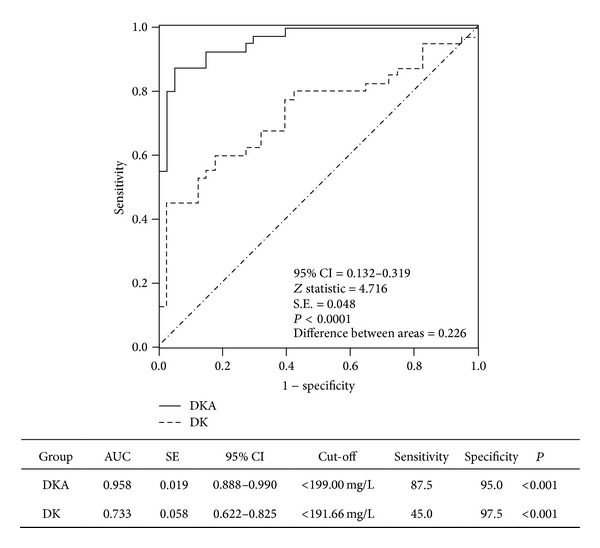
The ROC plots showed a significant difference in the ability of prealbumin levels to reflect DKA and DK. A comparison of the ROC plots between DKA and DK patients showed a significant difference (*P* < 0.0001).

The ROC curve for the comparison of prealbumin levels in DKA and DK patients is shown in Figure 2. Accordingly, area under the ROC curve (AUC) values were calculated and compared. There were no significant differences in the prealbumin levels of DKA and DK patients. To examine the predictive ability of prealbumin levels in estimating hyperglycemic crises, differences in the AUC values between DKA and DK were calculated, and significant differences were observed (*P* < 0.0001).

## 4. Discussion

The present study demonstrates that kidney-liver function is significantly altered in patients with DK and DKA. To our knowledge, there have been very few clinical studies on this topic. The data in the current study showed that in patients with ketoacidosis, the values of glycemic and renal variables were significantly increased, but the levels of protein biomarkers and eGFR were markedly reduced. The prealbumin levels were significantly reduced in hyperglycemic crisis. Although the exact mechanism behind this phenomenon is unclear, it can be concluded that the most significant factor influencing prealbumin levels might be metabolic acidosis status. Metabolic acidosis and ketone bodies induced an alteration in protein degradation and synthesis [15]. It is observed that cytokine and inflammatory mediators were increased, but the physiological protein were markedly reduced in patients with ketoacidosis [4, 5, 17, 19, 20]. Meanwhile, the risk factors caused an impairment of renal excretion. Insulin administration had a favorable effect on glucose levels and protein synthesis in the liver [16]. The diagnostic analysis presented here showed that lower prealbumin levels had a significant ability to reflect the presence of hyperglycemic emergencies. Prealbumin levels may be a novel marker for estimating hyperglycemic crisis. This study design limits the interpretation of the data because T1D and T2D have completely different pathophysiological mechanisms. However, kidney-liver function had a similar variation tendency in T1D and T2D subgroups. Although our results cannot replace traditional diagnostic criteria, this study adds complementary laboratory data by elucidating the patterns of kidney-liver function from stable condition to ketoacidosis.

Hyperketonemia with or without acidosis is considered to be an acute complication of poorly controlled or newly diagnosed diabetes mellitus. The degree of hyperketonemia severity is significantly associated with the arterial pH level. Higher H^+^ levels may significantly reduce the liver's production and release of enzymes and proteins [15]. In the present study, total protein, albumin, globulin, and prealbumin levels were significantly lower in the severe ketoacidosis cases compared with the mild/moderate cases. Elevated levels of ketone bodies can result in oxidative damage, accelerated apoptosis, and inhibition of cell growth in monocytes, which can lower the monocyte count [17]. We deduce that ketones may have a similar effect on hepatocytes. It is necessary for us to take future study.

In contrast to the levels observed at admission, the kidney function parameters recovered to the normal range, whereas the levels of proteins related to liver function increased after insulin treatment. A mild increase in AST and ALT levels is occasionally observed among patients with DK or DKA after insulin treatment, although the exact mechanism is still undefined [18]. It has been noted that DKA is associated with severe insulin deficiency, active systemic inflammatory processes, and oxidative stress [4, 19, 20]. Based on previous studies and our results, this clustered pathophysiological status may play a role in the development of kidney-liver function. Severe infection can aggravate the kidney-liver burden. In the ketosis patients, a decreasing tendency was observed between the noninfection and infection groups. However, no differences were found among the ketoacidosis patients. Mild infections can cause ketosis and ketoacidosis, although this was not the case in this study, indicating that the kidney and liver were not functioning well. The effect of DKA on kidney-liver function was far greater than the effect resulting from a mild infection.

The identification of prerenal deterioration has important clinical significance. Both ketosis and ketoacidosis are characterized by varying degrees of water and electrolyte depletion. A certain degree of prerenal azotemia can be associated with hyperglycemic emergencies. Dehydration leads to hypotension and a hyperosmolar state. Upon presentation of the symptoms, the magnitude of specific deficits in an individual patient varies, depending on the duration and severity of T1D or DKA [13, 21]. The success of treatment is significantly associated with the correct management of hyperglycemia reduction, metabolic acidosis rectification, and electrolyte deficit replacement [21]. Acute hyperglycemia causes vasodilation and alters endothelial function in adolescents with T1D [22]. Alterations of blood flow and endothelial function precede development of complications in T1D. Lowering blood glucose can reduce insulin resistance and oxidative stress and improve endothelial function in diabetes [23]. Insulin therapy is the cornerstone of treatment for DKA and DK, which can also result in an anti-inflammatory effect [24].

Biomarker discovery is known to be a valuable field of study and has become one of the most attractive subdisciplines in clinical proteomics for human diseases. Prealbumin, a visceral protein, is sensitive to protein malnutrition, and this biomarker has been detected in urine [25]. In the current study, prealbumin levels were positively correlated with the levels of HCO_3_ and arterial pH. However, prealbumin levels cannot be used to distinguish between infected and noninfected patients. Therefore, we can conclude that ketoacidosis induces changes in acute-phase response protein. Assessment of prealbumin levels will be helpful not only in early diagnosis but also in determining the prognosis of T2D patients. One limitation of this cross-sectional study is that the results represent preliminary evidence from a small study of patients with hyperglycemic emergencies. The clinical significance and the potential role of the alterations of prealbumin levels remain to be explored, particularly in diabetic patients with acute or chronic complications.

In summary, DK and DKA deteriorate liver and kidney function parameters, especially in patients with ketoacidosis. After considering the values of glycemic variables, the diagnosis of a hyperglycemic crisis should be cautious when based on lower prealbumin levels in overt hyperglycemia.

## Figures and Tables

**Table 1:** Clinical and biochemical characteristics of the study participants before and after treatment.

Variables	Controls (*n* = 40)	Stable patient (*n* = 40)	Total DK, before (*n* = 40)	Total DKA, before (*n* = 40)	Total DK, after (*n* = 40)	Total DKA, after (*n* = 40)
Female/male (*n*/*n*)	20/20	17/23	14/26	22/18	—	—
Age (years)	43 ± 8	49 ± 11	48 ± 16	43 ± 17	—	—
Diabetic type (1/2)	N/A	17/23	18/22	19/21	—	—
Duration of diabetes (years)	N/A	4.00 (0.00–20.00)	0.50^§^ (0.00–12.00)	5.50^†^ (0.00–26.00)	—	—
					—	—
Plasma glucose (mmol/L)	4.73 ± 0.33	10.37 ± 4.56**	16.69 ± 5.39^∗∗§§^	29.05 ± 7.98^∗∗§§††^	6.30 ± 1.02^*ϕϕ*^	7.39 ± 1.21^*ϕϕ*^
HbA1c (%)	5.20 ± 0.24	10.41 ± 2.58**	13.11 ± 2.64^∗∗§§^	12.50 ± 2.08^∗∗§§^	—	—
Alanine aminotransferase (U/L)	19.10 ± 5.86	21.95 ± 10.34	20.61 ± 10.72	15.74 ± 5.89^§^	24.80 ± 2.11^*ϕ*^	26.35 ± 1.87^*ϕϕ*^
Aspartate aminotransferase (U/L)	18.83 ± 4.20	19.13 ± 5.56	18.19 ± 5.71	19.04 ± 10.37	23.09 ± 2.44^*ϕ*^	24.37 ± 2.06^*ϕ*^
*γ*-glutamyl transpeptidase (U/L)	19.00 ± 9.16	30.24 ± 19.19*	21.47 ± 12.27^§^	17.96 ± 6.99^§^	37.07 ± 5.31^*ϕϕ*^	36.07 ± 4.25^*ϕϕ*^
Total bilirubin (*μ*mol/L)	13.45 ± 4.17	12.32 ± 4.72	14.33 ± 7.81	11.30 ± 4.54	11.85 ± 2.32	12.30 ± 1.55
Direct bilirubin (*μ*mol/L)	4.56 ± 1.51	4.68 ± 1.78	5.48 ± 3.20	4.62 ± 1.65	3.69 ± 1.54	4.09 ± 0.78
Total protein (g/L)	71.34 ± 3.39	70.66 ± 5.03	65.05 ± 6.52^∗∗§^	59.40 ± 9.47^∗∗§§†^	70.22 ± 4.41^*ϕϕ*^	69.87 ± 3.51^*ϕϕ*^
Albumin (g/L)	44.25 ± 2.18	43.69 ± 3.28	39.30 ± 5.16^∗∗§§^	35.99 ± 5.17^∗∗§§†^	41.39 ± 2.94^*ϕϕ*^	40.76 ± 1.06^*ϕϕ*^
Globulin (g/L)	27.10 ± 2.82	26.56 ± 3.84	25.99 ± 3.82	24.09 ± 6.46*	29.16 ± 2.44^*ϕ*^	29.50 ± 2.09^*ϕϕ*^
Prealbumin (mg/L)	270.67 ± 30.01	270.07 ± 62.71	212.90 ± 88.90^∗§^	137.84 ± 48.30^∗∗§§††^	267.85 ± 32.08^*ϕϕ*^	265.77 ± 29.91^*ϕϕ*^
Serum creatinine (*μ*mol/L)	63.90 ± 11.76	66.67 ± 12.80	61.21 ± 16.14	109.69 ± 74.77^∗∗§§††^	60.10 ± 5.54^*ϕϕ*^	46.59 ± 5.94^*ϕϕ*^
eGFR (mL/min per 1.73 m^2^)	105.97 ± 9.90	77.29 ± 14.54**	84.03 ± 18.85**	59.90 ± 25.68^∗∗§§††^	94.44 ± 18.53^*ϕϕ*^	92.80 ± 23.59^*ϕϕ*^
Uric acid (*μ*mol/L)	288.57 ± 75.09	260.34 ± 61.32	258.49 ± 99.63	467.22 ± 140.70^∗∗§§††^	230.54 ± 31.72^*ϕ*^	210.84 ± 49.62^*ϕϕ*^

Comparison with control group: *P < 0.05, **P < 0.001; comparison with stable DM group: ^§^ *P* < 0.05, ^§§^ *P* < 0.001; comparison with total DK group: ^†^ *P* < 0.05, ^††^ *P* < 0.001; comparison with pretreatment: ^*ϕ*^ *P* < 0.05, ^*ϕϕ*^ *P* < 0.001. All values in the table are given as the mean ± standard deviation, except for the duration of diabetes, which is given as the median and range (min–max).

**Table 2:** Clinical and biochemical characteristics of the T1D and T2D patients with DKA at admission.

Variables	T1D (*n* = 19)	T2D (*n* = 21)	*P* value
Female/male (*n*/*n*)	13/6	9/12	0.105
Age (years)	35 ± 19	52 ± 10	0.001
Duration of diabetes (years)	7.00 (0.00–26.00)	5.00 (0.00–15.00)	0.455
Plasma glucose (mmol/L)	31.82 ± 8.53	26.37 ± 6.81	0.035
HbA1c (%)	12.97 ± 2.25	12.07 ± 1.73	0.214
Arterial pH	7.01 ± 0.16	7.04 ± 0.14	0.486
HCO_3_ (mmol/L)	4.71 ± 3.50	5.54 ± 3.48	0.420
Alanine aminotransferase (U/L)	15.57 ± 5.79	15.92 ± 6.22	0.880
Aspartate aminotransferase (U/L)	19.21 ± 13.46	18.08 ± 5.98	0.782
*γ*-glutamyl transpeptidase (U/L)	17.31 ± 7.20	18.62 ± 6.99	0.643
Total bilirubin (*μ*mol/L)	11.42 ± 4.38	11.06 ± 4.71	0.843
Direct bilirubin (*μ*mol/L)	4.42 ± 1.27	4.78 ± 1.97	0.597
Total protein (g/L)	58.48 ± 11.60	60.32 ± 7.10	0.632
Albumin (g/L)	36.66 ± 4.76	35.42 ± 5.55	0.463
Globulin (g/L)	21.85 ± 6.94	26.48 ± 4.90	0.061
Prealbumin (mg/dL)	135.40 ± 52.78	130.53 ± 53.91	0.775
Serum creatinine (*μ*mol/L)	99.06 ± 53.62	119.32 ± 90.06	0.399
eGFR (mL/min per 1.73 m^2^)	62.90 ± 25.03	57.19 ± 26.58	0.490
Uric acid (*μ*mol/L)	458.53 ± 128.66	476.58 ± 157.40	0.746

All values in the table are given as the mean ± standard deviation, except for the duration of diabetes, which is given as the median and range (min–max).

**Table 3:** Basal clinical and biochemical characteristics of the DK and DKA patients with and without infection.

Variables	Noninfection DK (*n* = 26)	Infection DK (*n* = 14)	*P* value	Noninfection DKA (*n* = 21)	Infection DKA (*n* = 19)	*P* value
Female/male (*n*/*n*)	6/20	8/6	0.031	8/13	14/5	0.020
Age (years)	45 ± 16	55 ± 13	0.050	38 ± 16	50 ± 16	0.022
Duration of diabetes (years)	0.00 (0.00–8.00)	1.00 (0.00–12.00)	0.294	4.00 (0.00–26.00)	7.00 (0.00–18.00)	0.141
Plasma glucose (mmol/L)	17.62 ± 5.82	14.95 ± 4.13	0.136	32.05 ± 7.24	25.73 ± 7.58	0.010
HbA1c (%)	13.09 ± 3.19	13.15 ± 1.60	0.943	12.28 ± 2.31	12.65 ± 1.82	0.659
Arterial pH	7.37 ± 0.04	7.40 ± 0.05	0.142	7.03 ± 0.16	7.02 ± 0.13	0.747
HCO_3_ (mmol/L)	20.59 ± 5.13	22.49 ± 5.88	0.491	5.77 ± 4.26	4.56 ± 2.08	0.279
Alanine aminotransferase (U/L)	23.97 ± 11.33	13.92 ± 4.87	0.001	15.23 ± 6.07	16.21 ± 5.90	0.673
Aspartate aminotransferase (U/L)	18.79 ± 5.83	17.00 ± 5.49	0.382	19.77 ± 13.57	17.64 ± 6.51	0.604
*γ*-glutamyl transpeptidase (U/L)	21.38 ± 10.81	21.67 ± 15.31	0.948	16.42 ± 5.68	19.29 ± 7.91	0.306
Total bilirubin (*μ*mol/L)	15.59 ± 8.32	11.82 ± 6.23	0.175	11.02 ± 3.68	11.46 ± 5.20	0.806
Direct bilirubin (*μ*mol/L)	5.80 ± 3.39	4.83 ± 2.78	0.394	4.83 ± 1.65	4.37 ± 1.62	0.500
Total protein (g/L)	66.00 ± 6.90	63.14 ± 5.45	0.220	60.72 ± 12.59	58.27 ± 5.95	0.547
Albumin (g/L)	40.70 ± 5.16	36.68 ± 4.14	0.016	37.50 ± 5.43	34.42 ± 4.49	0.062
Globulin (g/L)	25.42 ± 3.83	27.13 ± 3.68	0.208	23.83 ± 7.40	24.46 ± 5.56	0.806
Prealbumin (mg/dL)	216.78 ± 74.33	204.81 ± 117.05	0.707	133.86 ± 43.96	142.85 ± 57.67	0.645
Serum creatinine (*μ*mol/L)	61.67 ± 17.22	59.44 ± 13.28	0.677	106.09 ± 80.06	110.86 ± 66.14	0.926
eGFR (mL/min per 1.73 m^2^)	87.31 ± 20.57	77.94 ± 13.81	0.136	63.11 ± 23.15	56.35 ± 28.44	0.413
Uric acid (*μ*mol/L)	256.73 ± 96.36	262.03 ± 110.22	0.883	483.52 ± 157.63	452.09 ± 127.05	0.572

All values in the table are given as the mean ± standard deviation, except for the duration of diabetes, which is given as the median and range (min–max).

**Table 4:** Clinical and biochemical characteristics of the mild/moderate and severe DKA patients at admission.

Variables	Mild/moderate DKA (*n* = 25)	Severe DKA (*n* = 15)	*P* value
Female/male (*n*/*n*)	15/10	7/8	0.517
Age (years)	48 ± 22	41 ± 14	0.198
Duration of diabetes (years)	8.00 (0.00–26.00)	5.00 (0.00–15.00)	0.134
Plasma glucose (mmol/L)	29.22 ± 9.24	28.94 ± 7.32	0.914
HbA1c (%)	12.35 ± 2.72	12.54 ± 1.72	0.837
Arterial pH	6.93 ± 0.10	7.18 ± 0.05	<0.001
HCO_3_ (mmol/L)	8.35 ± 3.17	3.27 ± 1.80	<0.001
Alanine aminotransferase (U/L)	14.37 ± 5.58	16.32 ± 6.06	0.445
Aspartate aminotransferase (U/L)	19.88 ± 7.47	18.16 ± 11.51	0.702
*γ*-glutamyl transpeptidase (U/L)	15.50 ± 3.96	19.06 ± 7.83	0.239
Total bilirubin (*μ*mol/L)	11.39 ± 3.39	11.19 ± 4.89	0.926
Direct bilirubin (*μ*mol/L)	4.34 ± 0.98	4.69 ± 1.82	0.641
Total protein (g/L)	58.81 ± 8.26	59.66 ± 10.18	0.838
Albumin (g/L)	37.07 ± 4.48	35.32 ± 5.54	0.309
Globulin (g/L)	23.39 ± 6.07	24.51 ± 6.60	0.686
Prealbumin (mg/dL)	171.94 ± 52.78	119.66 ± 35.44	0.010
Serum creatinine (*μ*mol/L)	117.44 ± 97.55	105.04 ± 58.94	0.618
eGFR (mL/min per 1.73 m^2^)	59.27 ± 25.91	60.28 ± 26.08	0.906
Uric acid (*μ*mol/L)	412.09 ± 168.84	490.44 ± 124.85	0.192

All values in the table are given as the mean ± standard deviation, except for the duration of diabetes, which is given as the median and range (min–max).
